# Congestion Analysis of Transport Layer in a Multicell 5G DL Communication System

**DOI:** 10.3390/s23136111

**Published:** 2023-07-03

**Authors:** Cristina Maria Andras, Gordana Barb, Florin Alexa, Cornel Balint

**Affiliations:** Department of Communications, Politehnica University of Timisoara, 300006 Timisoara, Romania; cristina.andras@student.upt.ro (C.M.A.); florin.alexa@upt.ro (F.A.); cornel.balint@upt.ro (C.B.)

**Keywords:** 5G communication systems, file transport protocol, transmission control protocol, slow-start algorithm

## Abstract

The fifth generation (5G) marks an important advance in mobile network capabilities. When it comes to high data rates, capacity, spectrum efficiency, and availability, 5G mobile broadband goes above and beyond what was previously possible with standard mobile broadband. The construction of 5G networks is still in the planning stages. These 5G networks will create intelligent networked communication environments by connecting people, things, data, applications, and transport networks. Mobile networks have made it possible for customers’ mobile devices (such as smartphones, tablets, laptops, and so on) to connect to the internet. A variety of distinct protocols will be required to take into consideration the various aspects that 5G possess. One of these is the transport protocol, which is intended to deliver extremely high data transfer rates up to 400 Gbps. The transmission control protocol (TCP) is one of the numerous protocols that are necessary for supporting 5G’s many capabilities. Our work focuses on the detection and analysis, on the downlink (DL) side, of the congestion of the transport layer in single- and multicell environments. For the purpose of the analysis, the following metrics were analyzed: physical resource blocks (PRBs), user throughput, cell throughput, cell edge user throughput, and delay. The work emphasizes the activation of the TCP slow-start algorithm using file transfer protocol (FTP) model two according to 3GPP standards.

## 1. Introduction

Wireless communication networks are making rapid progress towards their full implementation. It is already known that the complexity of fifth-generation (5G) systems will significantly increase, particularly in terms of the network architecture and wireless connection. These 5G systems bring new standards, increased energy and spectrum efficiency, extraordinarily high data rates, and new transmission techniques [1], while supporting a wide range of new applications and platforms, including the Internet of Things (IoT) [2], virtual reality (VR), augmented reality (AR) [3], autonomous vehicles, machine-to-machine (M2M) communications, tactile Internet (TI), software-defined networking, and multi-access edge computing. Optimizing the flow of traffic throughout the network and ensuring that several networks can coexist without interfering with one another is a crucial component in actual and future wireless communication networks [4,5]. The 5G technology has the potential to produce a transformative effect on multiple aspects of society by enhancing efficiency, productivity, and overall performance. The established goals of 5G are ambitious: capacity increases of up to a 1000 times, data rates of up to 20 Gbps, network slicing, and much higher stability [6].

The fifth generation is a substantial step forward in the capabilities of mobile networks. Up until this point, consumers’ mobile phones, tablets, and laptop computers have all been able to connect to the Internet thanks to the availability of mobile networks. When it comes to data rates, capacity, spectrum efficiency, and availability, 5G goes above and beyond what was previously possible with long-term evolution (LTE) and LTE Advanced. The implementation of 5G wireless communication demands the development of new use cases, new technologies, and new network architectures [7,8,9].

**Figure 1:**
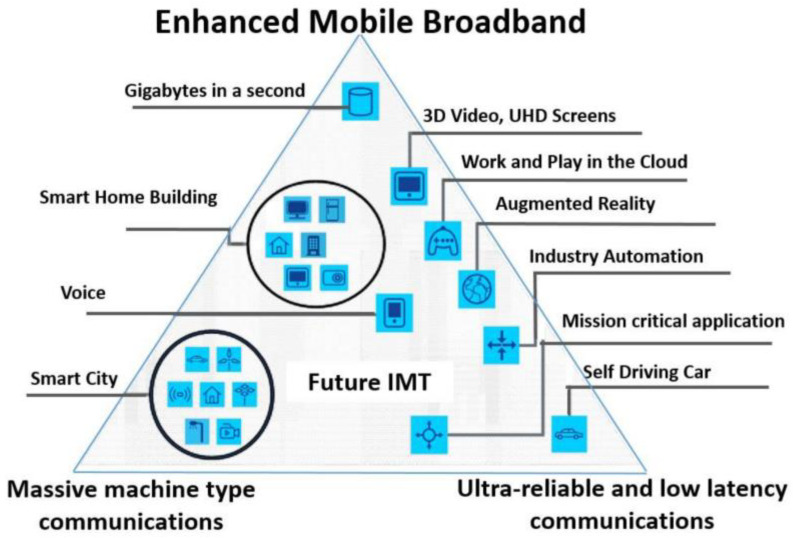
Fifth-generation (5G) use cases. Source: [22].

Through the development of the standard known as International Mobile Telecommunications (IMT), the International Telecommunication Union (ITU) has been actively participating in the process of worldwide standardization of mobile communication systems [10]. “IMT Vision—Framework and overall objectives of the future development of IMT for 2020 and beyond” was adopted as Recommendation ITU-R M.2083 [11] by the ITU in September of 2015. One of the most significant aspects of the Recommendation is the identification of three use-cases scenarios in IMT-2020 for 5G, see Figure 1, which are as follows:

- Enhanced mobile broadband (eMBB): Supporting eMBB usage scenarios, which demonstrate improved performance and a smoother user experience when compared to pre-existing mobile broadband applications, is a priority. Enhanced mobile broadband service presents high data speeds, high traffic capacity, and seamless mobility for hotspots and wide-area coverage [12]. The initial implementation of 5G networks will support consumer market needs, including rising smartphone subscriptions and mobile data growth. The 5G applications classified in this scenario are virtual reality, mobile cloud computing, fixed wireless, and UHD video. The authors of [13] underline that eMBB traffic uses the time slot as its transmission period to meet the requirements for both a high data rate and a high throughput. Essentially, eMBB service extends LTE Advanced broadband by allowing higher data rates and faster coding over large transmission blocks for a long duration. Thus, the eMBB system is targeted for high data rates and moderate reliability with a packet error rate (PER) of 10^−3^ [14]. The minimal peak data rates for the eMBB usage scenario are 20 Gbps for downlink (DL), 10 Gbps for uplink (UL), and 4 ms for user plane latency. For single-user downlink and uplink Internet protocol (IP) packet transfers, these performance requirements are expected;
- Ultra-reliable and low-latency communication (URLLC): The 5G technology must meet the stringent requirements for capabilities such as throughput, latency, and availability for applications such as wireless control of industrial production and manufacturing processes, remote medical surgery, distribution automation in smart grids, and transportation safety [15]. Notably, 5G release 15 established a basis for URLLC by incorporating new features, such as configurable sub-carrier spacing, a subslot-based transmission scheme, a new channel quality indicator, new modulation and coding scheme tables, and configured-grant transmission with automated repetitions [16]. Incoming URLLC users cannot be delayed to the next processing time slot due to severe delay limits. Hence, entering URLLC users pre-empt eMBB resources and begin transmission at the border of the next mini-slot after arriving [17]. Thus, URLLC can be used for public safety, intelligent transportation, remote healthcare, and industrial automation. These applications require ultra-low latencies, dependable communication, and high availability;
- Massive machine-type communication (mMTC): mMTC is a communication link that enables a high number of devices to speak with one another or with the Internet, with or without the involvement of humans, providing support for a very large number of connected devices, each of which normally sends a very low stream of data that is not time-sensitive [18]. Applications consist of sensor networks, smart homes, and smart cities. Machine-to-machine (M2M) communication is machine-centric communication between devices and sensors without human contact. Sensor networks under the IoT paradigm are driving machine-type communication with low-cost, low-power, and long-lasting sensors and devices. These devices and sensors normally transmit modest amounts of data that are not susceptible to delays. As the authors of [19] demonstrate, the primary problem is establishing massive communication lines for low-cost devices spread across vast regions while consuming ultra-low power.

Thus, 5G networks create intelligent networked communication environments by connecting people, data, applications, and transportation systems. It is going to require different protocols to accommodate their varied characteristics [20,21].

When thinking about mobile terrestrial access, one goal is to reduce delay as much as possible to less than 1 ms. As a result, there is ongoing research on the IP stack on the network layer. The TCP and user datagram protocol (UDP) are the two protocols that are used at the transport layer of networks [23]. TCP is built to disassemble a message into a series of data packets and to ensure that each packet properly reaches its destination as quickly as feasible, offering high data rates [24]. Hence, TCP is defined as a connection-oriented communication protocol that permits devices or applications to exchange data over a network and check their successfully delivery. UDP is a protocol that can be used instead of TCP; however, it does not offer error correction and its reliability is worse. Thus, the user datagram protocol (UDP) is a message-oriented communication protocol that allows devices and applications to traffic information via a network without verifying its delivery (incorrect sequencing cannot be detected or corrected), but it is the most suitable protocol for real-time communication (live data transmission), where the loss of a few packets is insignificant. Each of these variants was developed with the intention of using a congestion control method. Additionally, routing protocols can be added in order to increase the quality of service and the network’s performance, such as the destination sequence distance vector, Babel, dynamic source routing, Dymo, and the ad hoc on-demand distance vector [25].

Since UDP cannot control network congestion, this research focuses on TCP, which can run in a variety of different versions, each of which makes use of a congestion control algorithm. TCP is one of the many protocols that are needed to support the varied capabilities of 5G wireless communications [23]. When implementing either TCP or UDP in a mobile network, it is also important to take into account issues such as synchronization and functional slicing.

In this work, we studied the transport layer congestion in 5G communication systems by making use of two types of FTP traffic and the impact of the TCP slow-start algorithm. The effectiveness of TCP in a 5G network was evaluated using metrics such as user throughput (TP), cell throughput, cell edge user throughput, delay, and physical resource blocks (PRBs). Two types of FTP traffic were simulated to investigate the effect that the TCP slow-start model has on the levels of network congestion and delay. The goal of the TCP slow-start algorithm is to reach a suitable value for the congestion window as rapidly as possible.

It was observed that adopting the TCP slow-start method in either a single-cell or a multicell scenario can have a significant impact and offer many benefits. The obtained throughput was 1.2 Gbps for the single-cell scenario and 800 Mbps for the multicell scenario, keeping the maximum delay under 50 ms.

For the scenarios with greater amounts of UE in the cell, the cell edge user throughput decreased when the PRB utilization rate increased. Average throughput of 20 Mbps at the cell edge could be maintained up to around 38% PRB utilization in the cell for the multicell case, which was a similar value as obtained for the single-cell scenario. Up to about 40% of the cell’s PRBs could be used without negatively impacting the average throughput of 20 Mbps per second at the cell edge for the single-cell scenario.

## 2. Models and Methods

With regard to transport layers, as mentioned above, two main protocols have been designed: UDP and TCP. The TCP/IP model uses the network access layer to transfer data between two directly connected devices. The network access layer manages data transfer across the interface for 5G networks. TCP is used to decompose a message into data packets and to ensure that the message reaches its intended recipient as soon as possible [26]. TCP’s replacement, UDP, is less dependable and does not offer error correction. TCP, which has several different operating versions, is the subject of this research. These versions are all built with a congestion control mechanism.

Congestion control (CC) is an algorithm that limits the number of packets at network nodes. It works by restricting the available bandwidth. In this way, we can avoid overloading any node with an excessive quantity of packets [27]. A network is said to be congested when the rate of the packet flow towards any node is greater than its capacity to handle those packets. Congestion control can be implemented in a 5G network at either the network or transport layer, depending on the use case. It is reasonable to add the congestion control algorithm at the transport layer since this layer already facilitates the movement of data. One of the algorithms used is the slow-start algorithm, which is employed to prevent the need to wait for too many round trips for a large enough congestion window. When a node cannot handle the rate at which packets are flowing toward it, congestion occurs. In a 5G network, congestion control can be performed at either the network or transport layers. Since it provides for high-quality data delivery, congestion control should be included in the transport layer.

The main features of the CC algorithms are [28,29]:

- Preventing congestion collapse: The receiver can control the sender’s data flow using window-based flow control in the original TCP. The receiver’s data buffer space is protected by this flow control;
- High bandwidth utilization: CC algorithms optimize performance regarding throughput, delay, and loss. As the Web grows, Internet consumers desire high-bandwidth and low-delay communication rather than a slow background file transfer;
- Fairness: CC algorithms guarantee an acceptable, equal share of the available bandwidth among all competing flows sharing the same channel. TCP connections can share a congested link to distribute bandwidth fairly across similar flows;
- Fast convergence to fairness: CC algorithms are capable of rapid reactions to ensure the increase of the new flow and the reduction of the old flow until fairness is achieved. All flows need compatible congestion control techniques to share bandwidth equally;
- TCP-friendly: For deployment purposes, CC algorithms intended to be used in an uncontrolled network (e.g., the Internet) should coexist with other CC algorithms by maintaining fairness.

The TCP’s congestion control scheme holds the congestion window (CWND), which is in charge of limiting the amount of data transferred by the TCP in a single transmission interval [30,31]. It is crucial to remember that the transmission control block stores the current value of the CWND for each TCP connection. The best way to avoid congestion is to initiate the CWND since the sender is unaware of any network congestion that would prevent the data from reaching the receiver. The TCP connection must wait a long time between round trips before using the available bandwidth. The TCP congestion management technique, which involves round-trip incrementation of the CWND by a number of bytes equal to the maximum segment size (MSS), induces this situation.

The slow-start algorithm is used to avoid too much time being spent waiting for a large enough congestion window. The main goal of the TCP slow-start phase is to quickly arrive at a reasonable value for the congestion window [32,33]. The congestion window is doubled with every round trip during a slow-start process. The slow-start threshold is another variable utilized in TCP. This threshold is an estimate of the congestion window’s most recent value when the congestion process has failed to create it. The threshold must be changed during each congestion event after it is established at the sending window. When the window size value matches the slow-start threshold value, the slow-start process comes to an end. The next stage of congestion control then begins. If the user equipment (UE) does not initiate any congestion events, the slow-start procedure of the congestion control algorithm causes the congestion window to grow exponentially until the slow-start threshold is reached [34].

In wireless networks, packet loss is caused by transmission defects or other factors unrelated to congestion. TCP guarantees dependable transfers and can detect lost segments. There are two types of TCP congestion control scheme: mild congestion and severe congestion.

The authors of [35] emphasized that TCP considers the network to be lightly congested when it encounters mild congestion. Three duplicate acknowledgements are received in this case, resulting in quick retransmit. When only one segment is lost, the fast retransmit is considered a success. TCP performs a multiplicative decrease in this case, and the congestion window is divided by 2. This new congestion window value becomes the next slow-start threshold.

On the other hand, when the network’s retransmission timer expires, it becomes severely congested. The first segment is retransmitted by TCP, and the slow-start threshold is set to 50% of the congestion window. The TCP begins a slow-start process after the congestion window takes its initial value.

FTP refers to one set of rules that control how computers transfer files from one system to another via the Internet. The network protocol for transferring files between entities across TCP/IP connections was here assumed to be FTP. FTP is categorized as an application layer protocol as it uses the TCP/IP suite.

According to 3GPP TR 36.814 V9.2.0 [36], traffic models for system performance evaluations can be classified as full-buffer models, used for downlink and uplink continuous traffic, and non-full-buffer FTP models, used for downlink and uplink burst traffic. System throughput studies must be evaluated using a full-buffer traffic model that captures constant and non-varying traffic interference. Continuous traffic in the downlink and uplink uses the complete buffer model. On the other hand, the downlink and uplink burst traffic uses the non-full-buffer FTP model. The crucial factor in burst traffic is the duration of time that bursts are transferred for. This transfer time is influenced by the scheduling delay, the throughput, and the percentage of transmission time intervals in which the burst is scheduled. Additionally, burst traffic models must be used for evaluations with time-varying interference.

There are two different FTP traffic models under the heading of non-full-buffer models: FTP model one and FTP model two. The file size for FTP traffic model one is currently set to 2 Mbytes; however a size of 0.5 Mbytes can be used to accelerate simulations. Using a Poisson distribution and an arrival rate of *λ*, the number of users, denoted K, can be computed. According to the 3GPP standard mentioned above, the possible ranges for the arrival rate are as follows: [0.5, 1, 1.5, 2, 2.5] for a file size of 0.5 Mbytes and [0.12, 0.25, 0.37, 0.5, 0.625] for a file size of 2 Mbytes.

**Figure 2:**
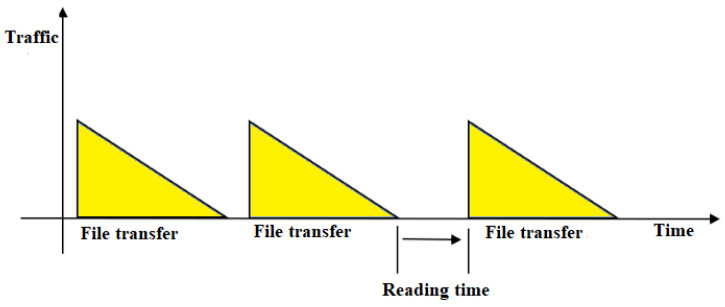
FTP traffic model two.

The user arrival time with FTP traffic model two, referred to as a general traffic model, which corresponds to most circumstances, is shown in Figure 2.

The number of users is fixed at two in FTP model two, and the file size is set to 0.5 Mbytes. The reading time *(d)* is a random variable that has an exponential distribution with a mean value of five seconds (rate *λ* = 0.2). The probability density function of *d* can be estimated as follows:

*f_d_* = *λe*^−*λd*^, *d* ≥ 0, *λ* = 0.2 (1)

## 3. Experiments

This section presents the setup parameters for the simulations, as well as the environment scenarios selected. As presented in Table 1, we focused on downlink simulations for the two following environments: a single-cell environment and a multicell environment consisting of three cells.

**Figure 3:**
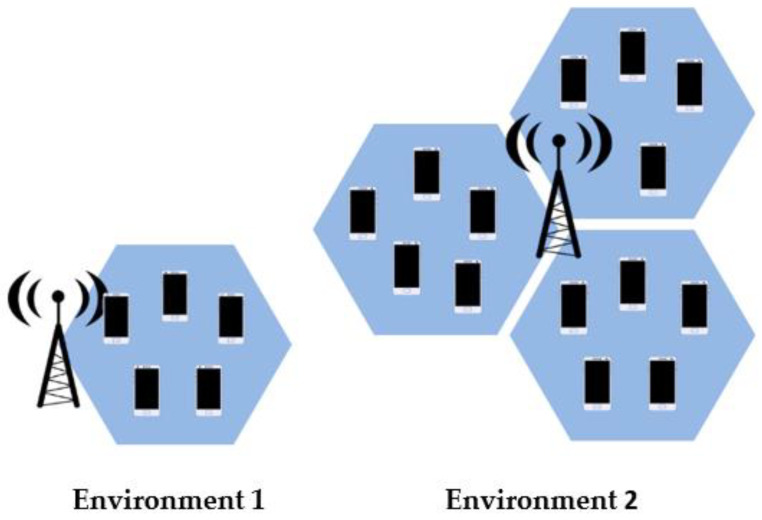
Graphical representation of environments one and two, respectively.

The channel model adopted was an urban macrocell environment without buildings, where the gNodeB (gNB) had 64 transmitter antennas and 64 receiver antennas, respectively. We considered 10 to 200 UE devices, depending on the scenario simulated, which moved at a walking speed of 3 km/h (see Figure 3 for a comparison of both environments tested).

The configuration implemented consisted of a 5G standalone (SA) architecture, option two, with the core and radio components of the architecture using 5G interfaces and features.

The deployment type selected was time-division duplexing (TDD) with a carrier frequency of 3.5 GHz, as this is a typical range for frequencies allocated for 5G networks, and a bandwidth of 100 MHz, allowing for higher data rates and increased network capacity. The modulation adopted was 256 quadrature amplitude modulation (QAM) and the MIMO mode was 4 × 4 MIMO with grid-of-beams (GoB) digital beamforming implemented. This allowed the exploitation of spatial diversity, increased data rates, and optimization of coverage by dynamically adapting beam patterns. The test environment was formed by an urban macrocell (UMa) environment without buildings, where the users moved at a walking speed of 3 km/h. To perform the simulations, we used Nokia’s internal 5G simulator 5GMax alongside the MATLAB program. See Table 2 for the detailed list of parameters adopted.

Our intention was to detect and investigate the congestion that occurs in a 5G network on the DL side. The traffic model used for all simulations was FTP model two with the TCP slow-start algorithm; furthermore, 10 extended files of 5 Mbytes each, a duration of 5 s, and geometric distribution were employed in order to create enough PRB utilization and achieve divergent loading in the downlink. The slow-start algorithm was hence activated to investigate its impact on 5G network congestion and delays. This means that a single 1500-byte packet was sent into the buffer; however, several smaller packets were continuously increased until an optimal value was found.

The metrics studied for this work were as follows: PRB utilization, for which all slots were included, regardless of whether there was traffic or not; data volume, defining the average volume of data waiting in the buffer; cell throughput, representing the average throughput of the cell; cell edge user throughput, consisting of the 5% tile of the user throughput distribution; user throughput; and delay, for which all slots were included, regardless of whether there was traffic or not. The user throughput was calculated as:

(2) $$\bar{{TP}_{user}}=\frac{packet\_size}{time\;of\;transmission\;+\;time\;waiting\;in\;buffer}$$

Table 3 presents the scenarios and metrics simulated for each environment.

## 4. Discussion

This section presents the simulation results and discussion according to the simulation environment using the scenarios defined in Table 3.

**Single-cell environment:**

**Figure 4:**
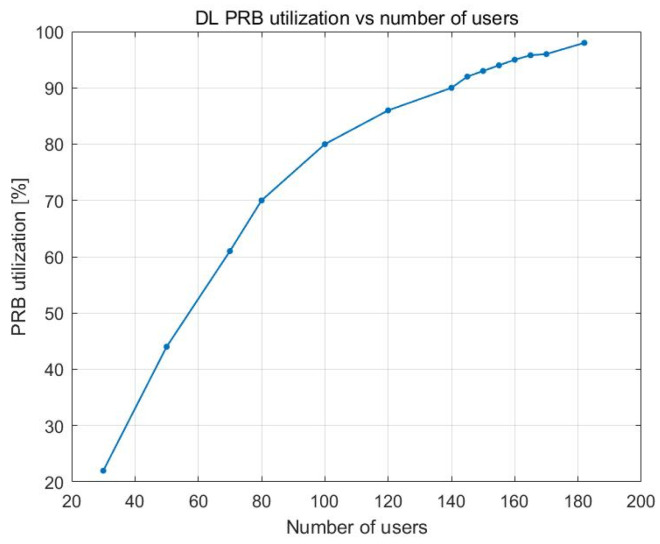
DL PRB utilization vs. number of users.

Figure 4 shows the impact of the number of users on the utilization of the downlink PRBs. After the PRB utilization rate reached approximately 70% with 80 UE devices, its variation became nonlinear and, at approximately 140 UE devices, it began to level off with a 100% PRB utilization rate.

**Figure 5:**
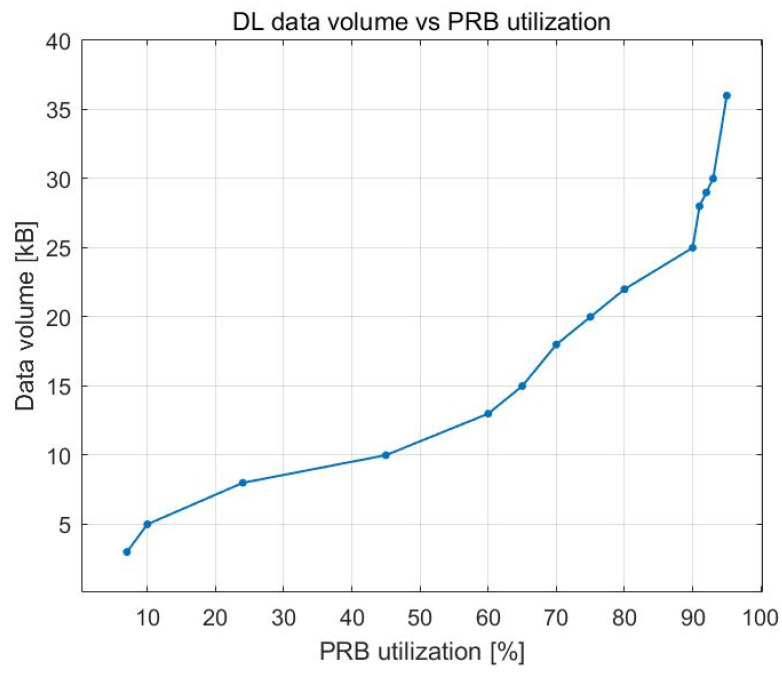
DL data volume vs. PRB utilization.

The data volume, which, in this context, refers to the data waiting in the buffer to be sent, was relatively stable up to approximately 80–90% PRB utilization, as presented in Figure 5. After that point, it started to rapidly increase because, as the number of UE devices increased, the amount of data that had to be delivered by the gNB also increased; however, there were no free PRBs.

**Figure 6:**
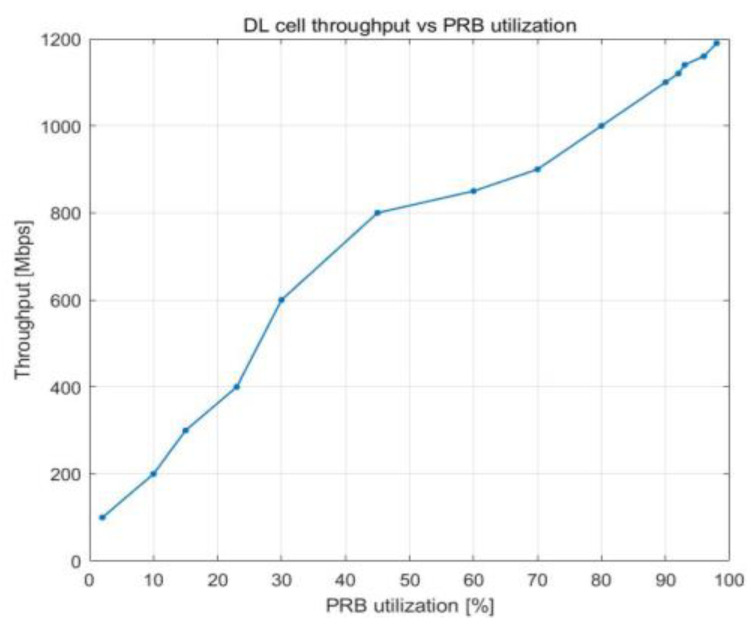
DL cell throughput vs. PRB utilization.

**Figure 7:**
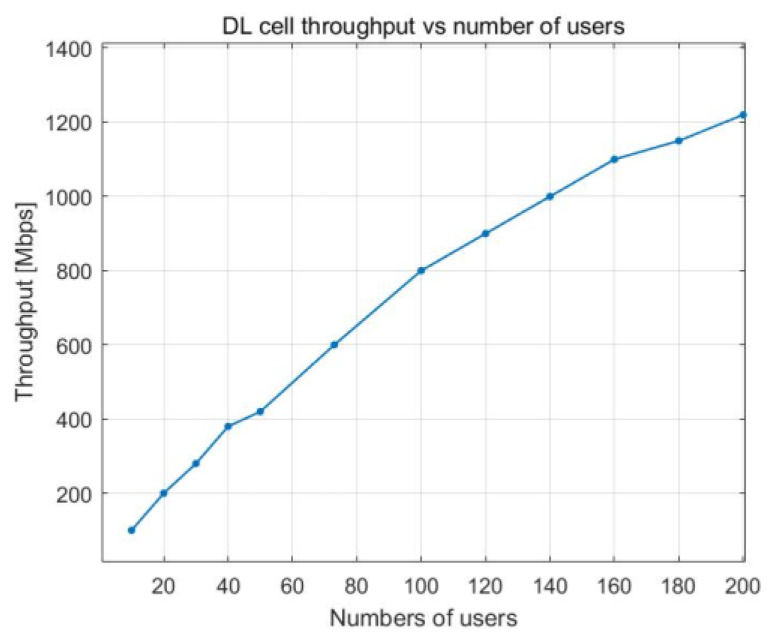
DL cell throughput vs. number of users.

The charts in Figure 6 and Figure 7 show how the cell throughput shifts when the number of users in the cell and the PRB utilization both increase. The cell throughput in the downlink direction shows linear growth. In Figure 6, we can observe that the maximum throughput for a cell is 1.2 Gbps. The throughput achieved is extremely high because of additional capabilities, such as 4x4 MIMO and 256 QAM modulation. The cell throughput is 800 Mbps when there are 100 UE devices. When the number of users in a cell doubles, the throughput of the cell increases by approximately 50%, reaching a maximum of 1.2 Gbps (see Figure 7).

**Figure 8:**
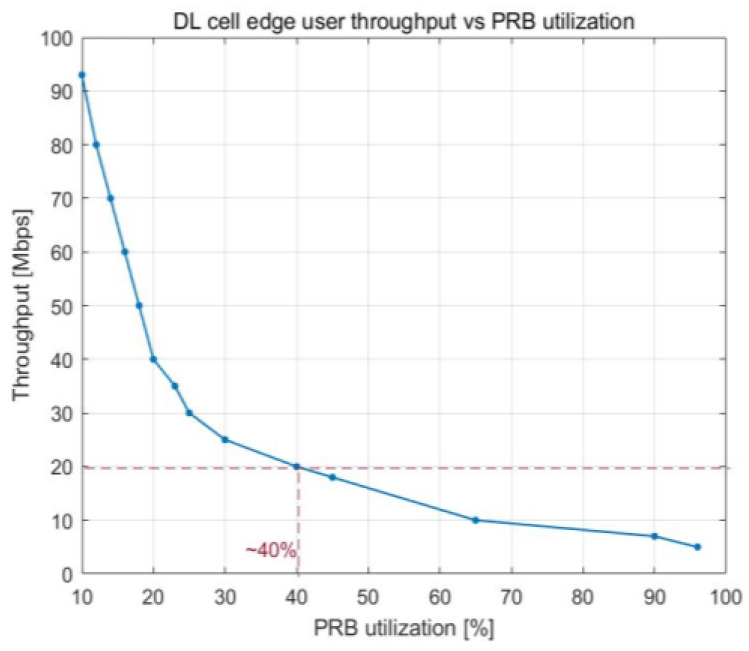
DL cell edge user throughput vs. PRB utilization.

**Figure 9:**
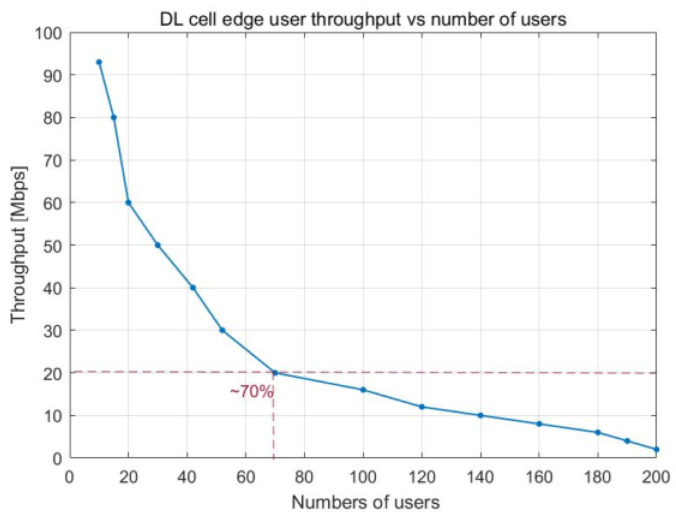
DL cell edge user throughput vs. number of users.

The changes in the cell edge user throughput in response to the number of PRBs and the total number of cell users are emphasized in Figure 8 and Figure 9, respectively. As the number of UE devices in the cell increases, the cell edge user throughput available decreases due to the increase in PRB utilization. An average throughput of 20 Mbps at the cell edge can be observed with approximately 40% PRB utilization, which is equivalent to approximately 70 UE devices in the cell. After this point, the cell edge user throughput is extremely low.

**Figure 10:**
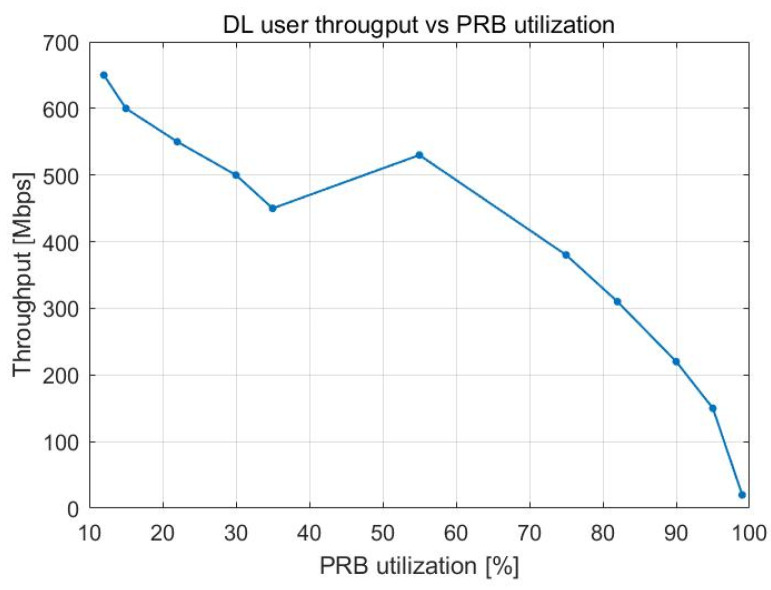
DL user throughput vs. PRB utilization.

**Figure 11:**
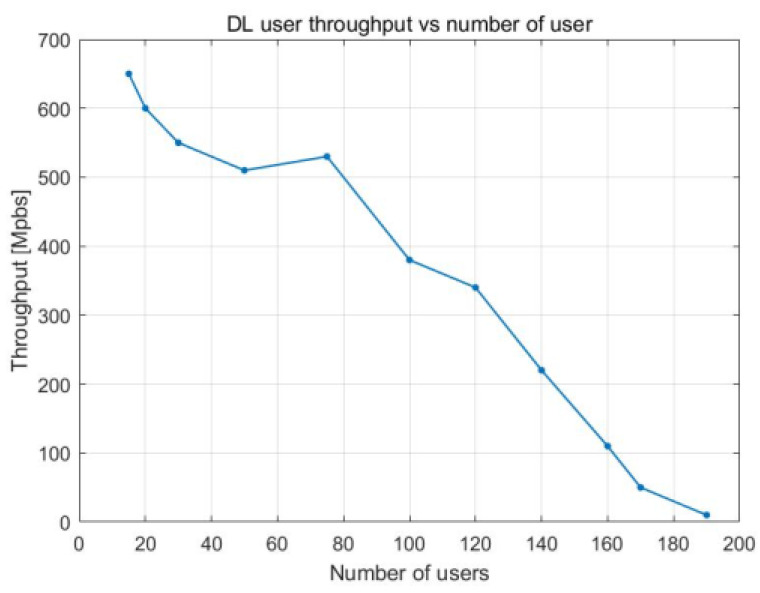
DL user throughput vs. number of users.

User throughput decreases with the increase in PRB utilization as there are more UE devices in the cell. This trend can be seen in Figure 10 and Figure 11, which present how the relationship between the two variables evolves over time. Between 10 and 50% PRB utilization, the user throughput is between 650 Mbps and 500 Mbps, respectively. The decline in user throughput follows a linear pattern, with a more noticeable incline after 50% load in the cell. A maximum user throughput of 650 Mbps is achieved when there are 20 UE devices. This value drastically falls to half (i.e., 375 Mbps) when there are 100 UE devices. From 160 UE devices onward, the user throughput is at its lowest.

**Figure 12:**
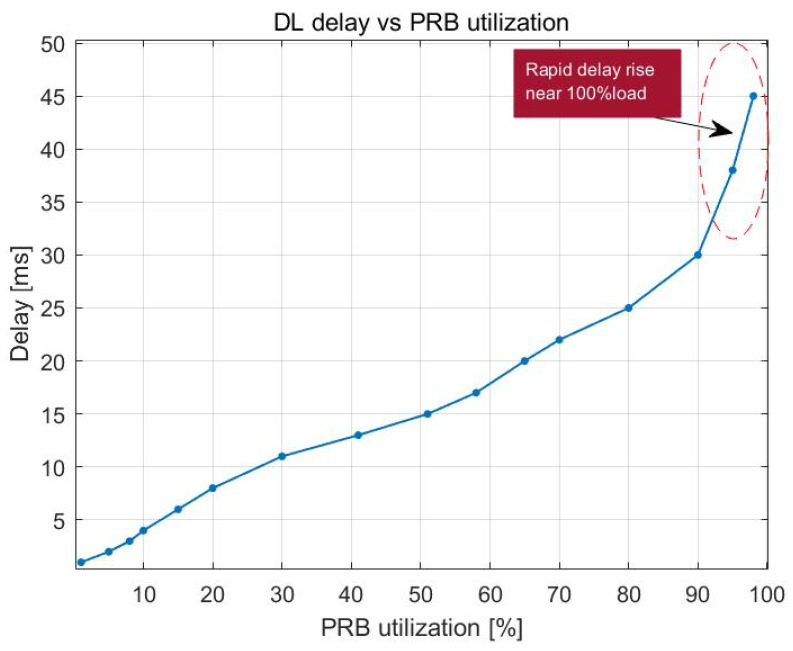
DL delay vs. PRB utilization.

**Figure 13:**
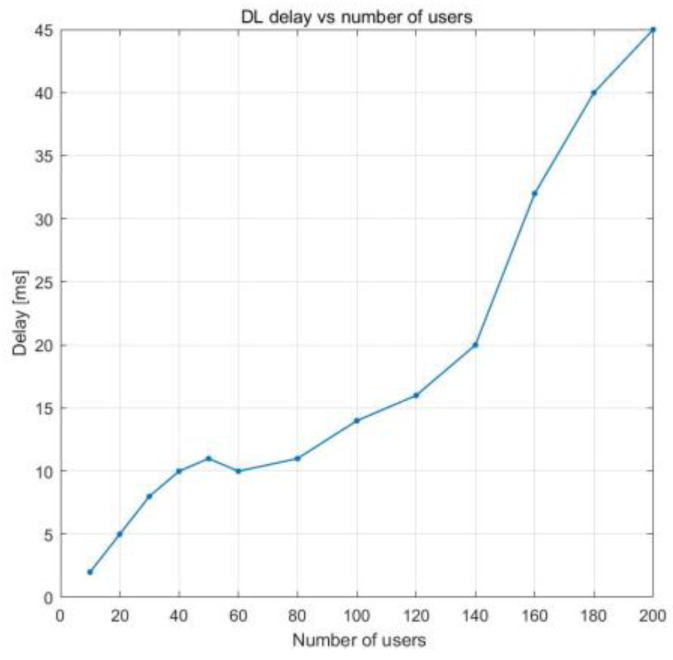
DL delay vs. number of users.

Figure 12 and Figure 13 indicate how latency in the downlink changes as the number of UE devices in the cell and the use of PRBs increase. The delay gradually increases up to roughly 98% PRB utilization in the cell, at which point it begins to increase exponentially, reaching a maximum of 45 ms at 100% PRB utilization. The delay begins to increase when the cell has approximately 140 UE devices, which indicates that, with that number of UE devices, there is already a load that is close to 100% PRB utilization.

**Multicell environment:**

**Figure 14:**
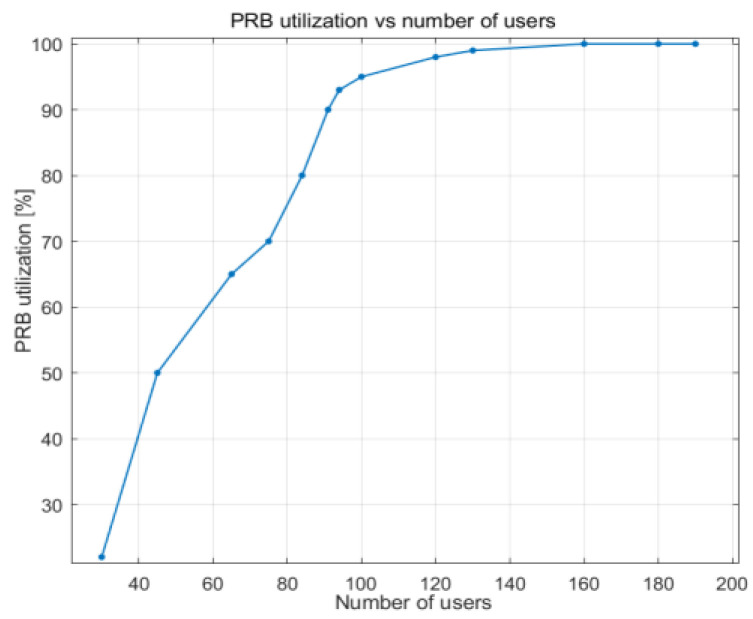
PRB utilization vs. number of users.

This section presents the results obtained with the multicell scenario, where we simulated three cells. Figure 14 presents the PRB utilization according to the number of users. It can be observed that, at 45 UE devices, the PRB utilization is 50%, and it increases linearly until there are approximately 100 UE devices, at which point it saturates at around 100% PRB usage. 

**Figure 15:**
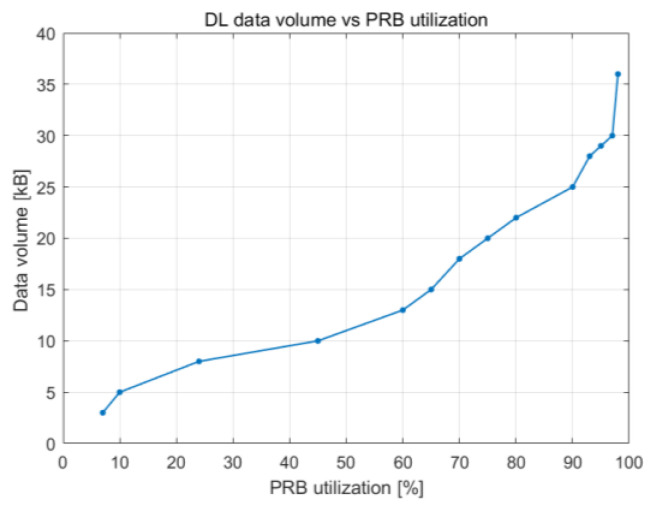
DL data volume vs. PRB utilization.

Data volume represents the data waiting to be transmitted in the buffer. As shown in Figure 15, data volume steadily increases up to approximately 90% PRB utilization. As the number of UE devices increases, the amount of data that needs to be sent by the gNB increases, but since there are no available PRBs, more data remain in the buffer.

**Figure 16:**
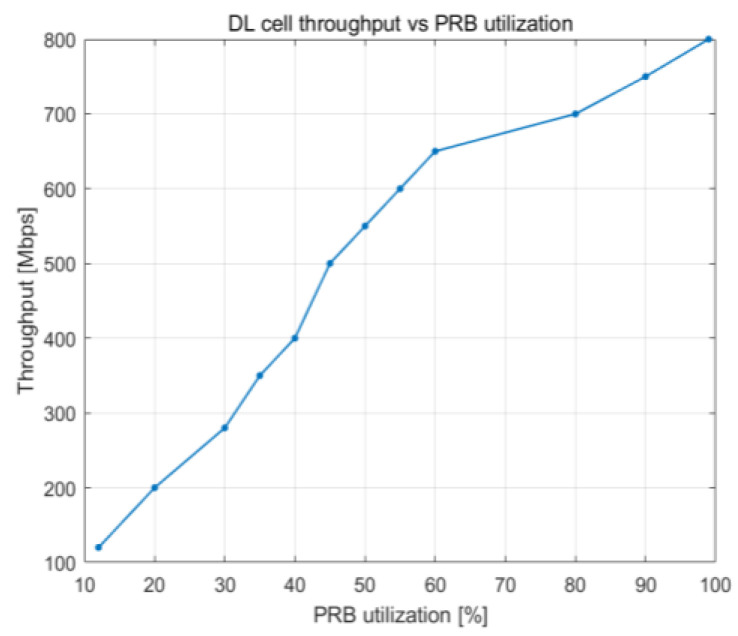
DL cell throughput vs. PRB utilization.

**Figure 17:**
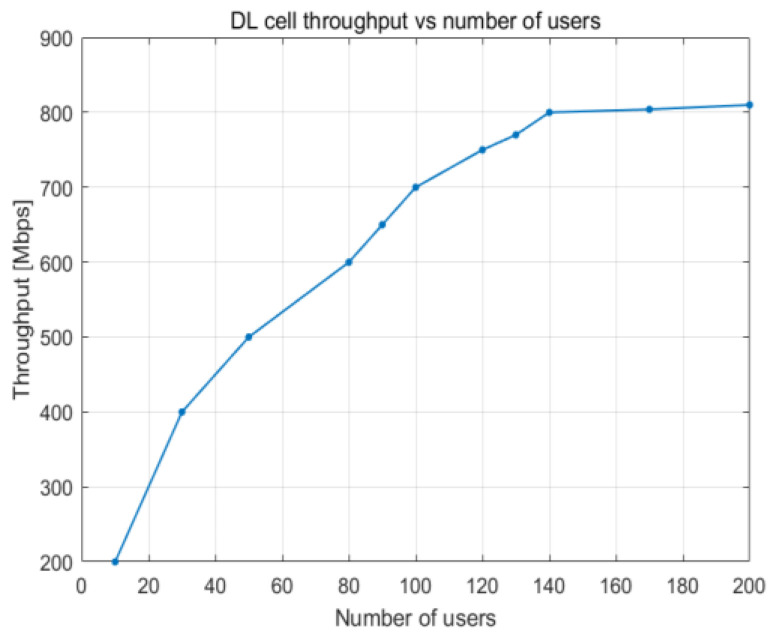
DL cell throughput vs. number of users.

The relationship between the utilization of PRBs and the number of users working in the cell is illustrated in Figure 16, which depicts how cell throughput shifts over time. Cell throughput increases linearly until there are 100 UE devices in the cell, reaching a maximum of approximately 800 Mbps. The variation in cell edge user throughput that results from an increase in PRB utilization is illustrated in Figure 17. Cell edge user throughput drops with the increase in PRB utilization since there are more UE devices in the cell. This causes a decrease in overall cell throughput. It is possible to sustain an average throughput of 20 Mbps at the cell edge with PRB utilization of approximately 38%.

**Figure 18:**
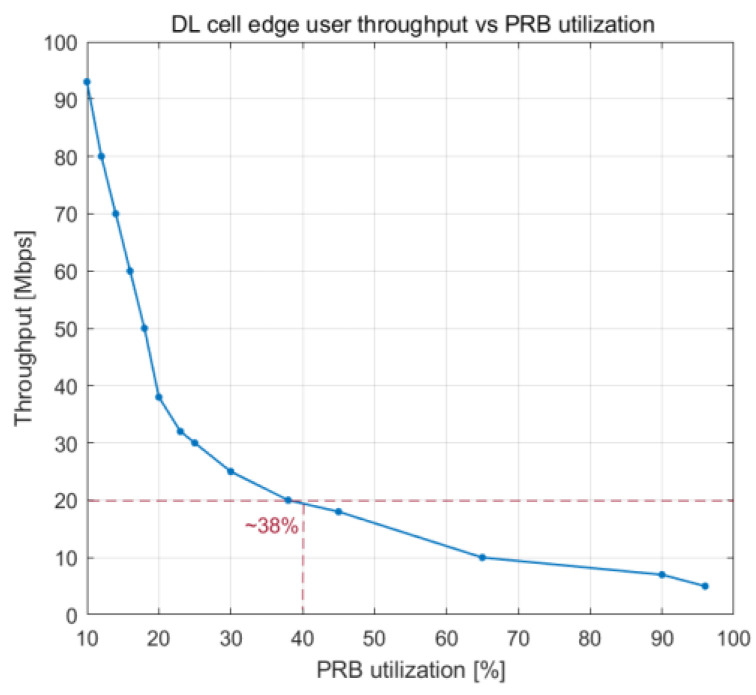
DL cell edge user throughput vs. PRB utilization.

**Figure 19:**
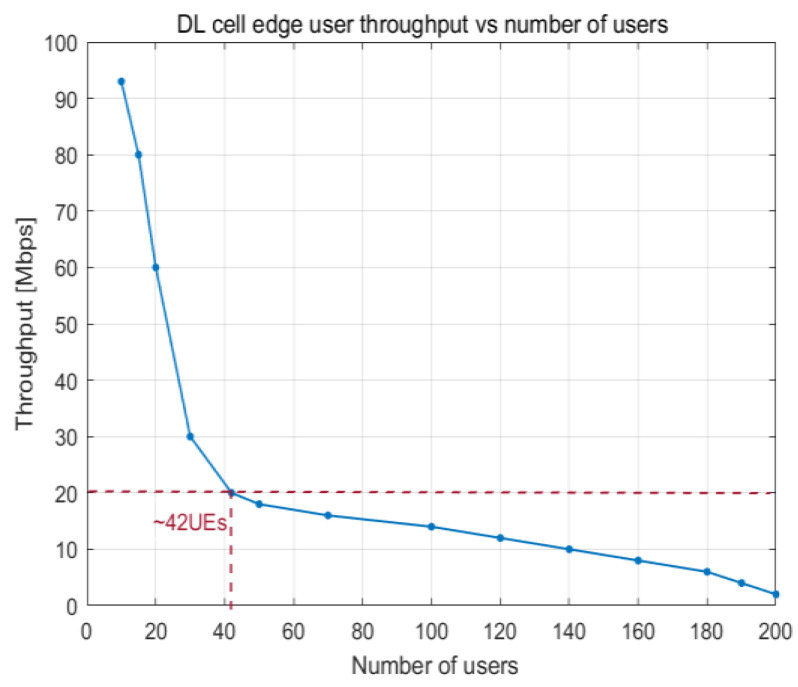
DL cell edge user throughput vs. number of users.

Figure 18 and Figure 19 show how the PRB utilization and number of users influence the throughput experienced at the edge of a cell, respectively. When there are around 42 UE devices at the cell edge, a throughput of 20 Mbps is achieved on average. The decrease in cell edge throughput has a substantial impact on the communication link when the number of users exceeds that threshold.

**Figure 20:**
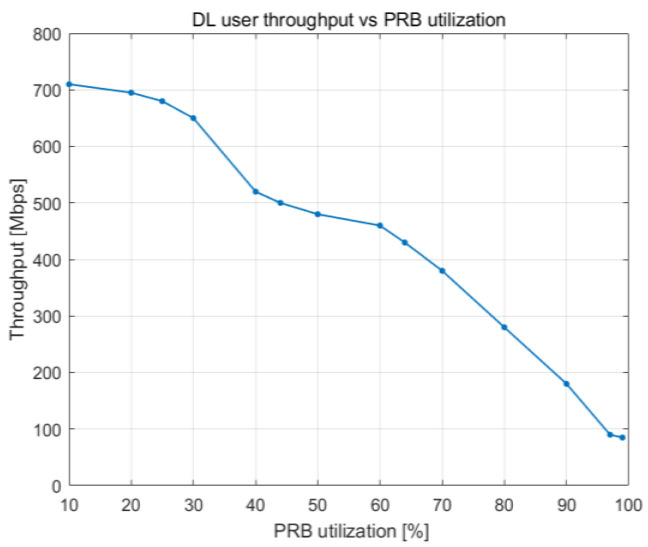
DL user throughput vs. DL PRB utilization.

**Figure 21:**
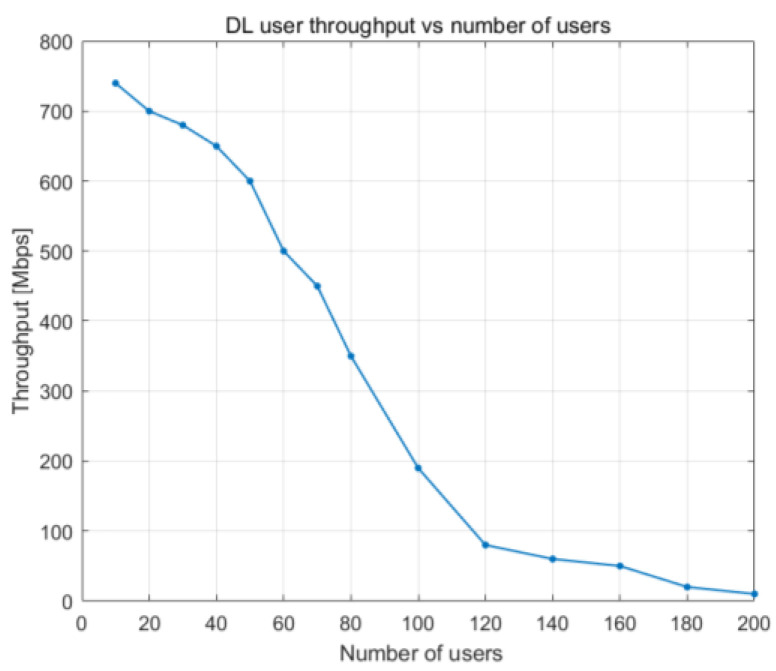
DL user throughput vs. number of users.

Figure 20 and Figure 21 illustrate how user throughput shifts when the percentage of PRBs being utilized and the total number of users in the cells rise, respectively. User throughput drops as the number of UE devices and the PRB utilization increase. The decrease follows a linear pattern, and it becomes more noticeable when the cell is loaded to between 60 and 100% of its capacity.

In Figure 20, we can observe a maximum user throughput of 700 Mbps when there are 20 UE devices. This value drastically falls to half (i.e., 350 Mbps) when there are 80 UE devices. From 120 UE devices onward, the user throughput is at its lowest. 

**Figure 22:**
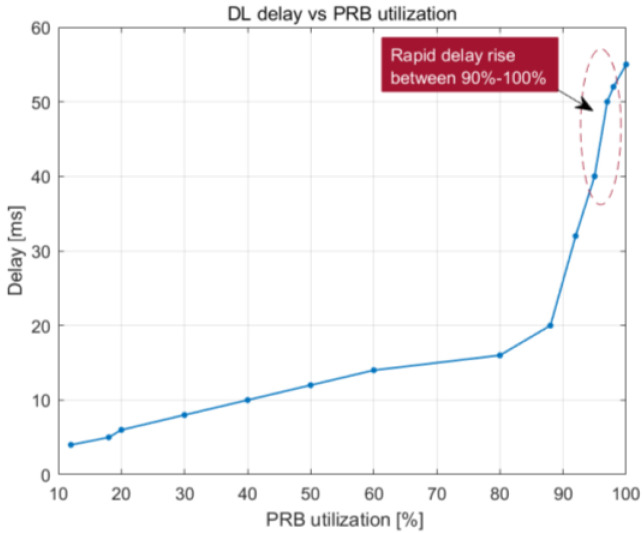
DL delay vs. PRB utilization.

**Figure 23:**
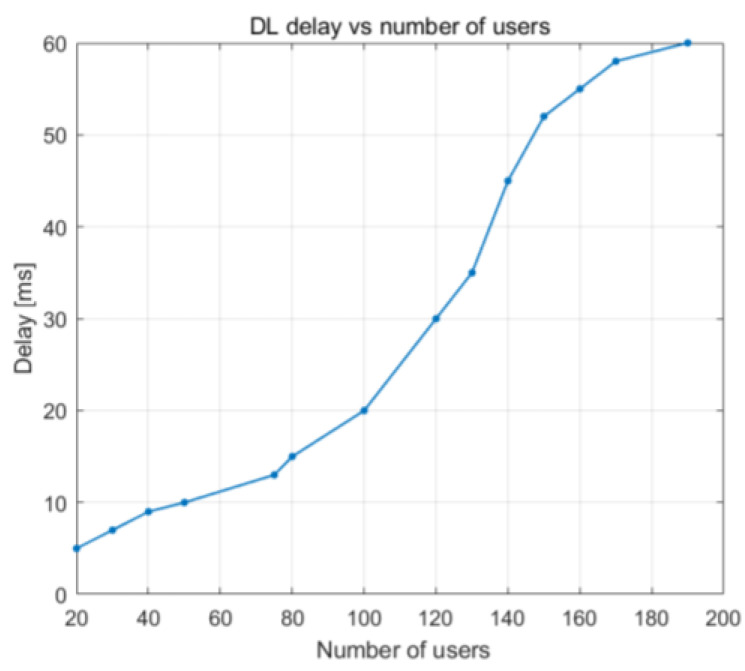
DL delay vs. number of users.

Figure 22 and Figure 23 illustrate how the downlink latency shifts when the percentage of PRBs being used and the number of UE devices increase. The delay steadily increases up to around 93% PRB utilization, at which point it is approximately 20 ms. After that, it begins to rise at a faster rate as the buffer reaches its 100% capacity, leading to an increasing number of packets that need to wait in the buffer before being broadcast. The load becomes close to 100% PRB utilization when there are 80 or more UE devices, which explains why delay begins to grow at that time. The average latency decreases to 10 ms once there are 70 UE devices or less.

Table 4 provides a comparison of the metrics and results obtained using the single-cell and multicell environments.

The results obtained in both environments simulated are of great importance and provide insightful information on how the 5G network behaves when dealing with congestion.

The simulations showed that DL data volume was stable up to 80–90% PRB usage and the maximum throughput was about 1.2 Gbps in a single-cell scenario and 800 Mbps in a multicell scenario. An average throughput of 20 Mbps for the cell edge users was observed and the maximum delay was 45 ms for a single-cell scenario and 55 ms for multicell scenario.

## 5. Conclusions

In this paper, we analyzed how the 5G network behaves when dealing with congestion. It was underlined that adopting the TCP slow-start method in either a single-cell or a multicell scenario can have a significant impact and, additionally, offer many benefits (e.g., throughput up to 1.2 Gbps for a single-cell scenario vs. 800 Mbps for a multicell scenario, keeping the maximum delay under 50 ms, etc.). The parameters that were investigated were PRB utilization, data volume, delay, cell throughput, and average cell user throughput.

We adopted FTP model two for all simulations performed. To obtain sufficient PRB utilization and divergent loading in the DL, the TCP slow-start algorithm was activated using 10 extended files of 5 Mbytes each, a length of 5 s, and geometric distribution. The slow-start algorithm was activated to ensure its influence on the congestion and delays in the 5G network. A single packet with a size of 1500 bytes was loaded into the buffer; however, the sizes of several smaller packets were incrementally raised until a value that was considered effective was obtained. The average user throughput was calculated by averaging the packet size over the transmission duration, including buffer waiting time. In the DL scenarios, these performance parameters were shown as a function of PRB utilization and the number of users.

Given a greater number of UE devices in the cell, the cell edge user throughput decreased when the PRB utilization rate increased. Average throughput of 20 Mbps at the cell edge could be maintained up to around 38% PRB utilization in the cell for the multicell case, which was a similar value as that obtained for the single-cell scenario. Up to about 40% of the cell’s PRBs could be used without negatively impacting the average throughput of 20 Mbps at the cell edge for the single-cell scenario. The cell throughput was more linear since more UE devices with larger files were simulated, generating more samples. The delays in the downlink direction for the multicell scenario were maintained at a low level (10 ms) up to approximately 45% PRB use; for the single-cell scenario, they were maintained at this level up to approximately 50% PRB utilization.

Networks’ focus shifts with each generation of communication technology. The next generation is the sixth generation (6G), intended to boost every network connectivity upgrade that 5G can currently provide to users. Smart cities, farms, industries, and robotics will improve with 6G. For several technological and use-case aspects, 6G will build on 5G, driving its adoption at scale through optimization and cost-cutting. Simultaneously, 6G will enable new applications.

## Figures and Tables

**Table 1:** Environments simulated in this work.

**Environment one**	Single-cell	One cell
**Environment two**	Multicell	Three cells

**Table 2:** List of parameters.

Parameter	Values
Duplex mode	TDD
Channel bandwidth	100 MHz
Frequency	3.5 GHz
Type of scenario	Multicell
Channel model	UMa without buildings
gNB antenna configuration	64T64R
gNB Tx power	200 W
DL MIMO mode	4 × 4 MIMO
Beamforming	GoB beamforming
Number of UE devices per cell	From 10 to 200
UE speed	3 km/h
Maximum UE power	23 dB
Traffic model	FTP model two: extended 10 × 5 MB file with TCP slow-start algorithm
Modulation	DL: 256QAM
Simulation length	30 s

**Table 3:** Scenarios and metrics investigated.

Scenarios	Metrics Studied
Scenario one	PRB utilization and data volume
Scenario two	Cell throughput
Scenario three	Cell edge throughput
Scenario four	User throughput
Scenario five	Delay

**Table 4:** Comparison of the results obtained.

Metric Evaluated	Single-Cell	Multicell
DL PRB utilization vs. number of users	50% PRB use with 58 UE devices 100% PRB use with 140 UE devices	50% PRB use with 45 UE devices 100% PRB use with 100 UE devices
DL data volume vs. PRB utilization	Stable until 80–90% PRB use	Stable until 90% PRB use
DL cell throughput vs. PRB utilization	Maximum TP speed obtained was 1.2 Gbps	Maximum TP speed obtained was 800 Mbps
DL cell throughput vs. number of users	With 100 UE devices, 800 Mbps TP speed With 200 UE devices, there was an increase of 50% in the TP speed (1.2 Gbps)	With 100 UE devices, 750 Mbps TP speed With 200 UE devices, there was an increase of 7% TP in the TP speed (800 Mbps)
DL cell edge user throughput vs. PRB utilization	Average of 20 Mbps with 40% PRB utilization	Average of 20 Mbps with 38% PRB utilization
DL cell edge user throughput vs. number of users	Average of 20 Mbps with 20 UE devices	Average of 20 Mbps with 42 UE devices
DL user throughput vs. PRB utilization	With 50% PRB use, 500 Mbps	With 50% PRB use, 480 Mbps
DL user throughput vs. number of users	With 100 UE devices, 375 Mbps	With 100 UE devices, 190 Mbps
DL delay vs. PRB utilization	Maximum delay of 45 ms with 100% PRB use	Maximum delay of 55 ms with 100% PRB use
DL delay vs. number of users	Delay increased rapidly with >140 UE devices	Delay increased rapidly with >140 UE devices

## Data Availability

Data sharing not applicable.

## Abbreviations

5G	fifth generation
6G	sixth generation
CC	congestion control
CWND	congestion window
DL	downlink
eMBB	enhanced mobile broadband
FTP	file transfer protocol
GoB	grid of beams
IoT	Internet of Things
IP	Internet protocol
ISD	intersite distance
LTE	long-term evolution
M2M	machine-to-machine
MIMO	multiple-input, multiple-output
mMTC	massive machine-type communication
MSS	maximum segment size
PER	packet error rate
PRB	physical resource block
QAM	quadrature amplitude modulation
SA	standalone
TCP	transmission control protocol
TDD	time-division duplex
TI	tactile Internet
TP	user throughput
UDP	user datagram protocol
UE	user equipment
UMa	urban macrocell
URLLC	ultra-reliable and low-latency communication
